# Can Brain Health Be Supported by Vitamin D-Based Supplements? A Critical Review

**DOI:** 10.3390/brainsci10090660

**Published:** 2020-09-22

**Authors:** Mahitab Farghali, Sara Ruga, Vera Morsanuto, Francesca Uberti

**Affiliations:** Laboratory of Physiology, Department of Translational Medicine, University of Piemonte Orientale, Via Solaroli 17, 28100 Novara, Italy; sara.ruga@uniupo.it (S.R.); vera.morsanuto@med.uniupo.it (V.M.)

**Keywords:** vitamin D, brain aging, neurodegenerative diseases, neuroinflammation, VDR

## Abstract

This review presents recent knowledge on the neuroprotective effects of vitamin D and their usefulness as oral supplementation when combined with other molecules, such as curcumin. A critical look at the effectiveness of vitamin D in this field is also provided. Vitamin D plays a crucial role in neuroprotection and in the cognitive decline associated with aging, where vitamin D’s levels are related to the levels of several neurotrophic factors. An important role of vitamin D has also been observed in the mechanism of neuroinflammation, which is the basis of several aging conditions, including cognitive decline and neurodegeration; furthermore, the neuroprotective effect of vitamin D in the cognitive decline of aging has recently been reported. For this reason, many food supplements created for humans contain vitamin D alone or combined with other molecules with antioxidant properties. However, recent studies also explored negative consequences of the use at a high dosage of vitamin D. Vitamin D in tissues or brain cells can also modulate calbindin-D28K, parvalbumin, and calretinin, and is involved in immune function, thanks also to the combination with curcumin. Curcumin acts as a free radical scavenger and antioxidant, inhibiting lipid peroxidation and oxidative DNA damage. In particular, curcumin is a potent immune-regulatory agent and its administration has been reported to attenuate cognitive impairments. These effects could be exploited in the future to control the mechanisms that lead to the brain decay typical of neurodegenerative diseases.

In recent years it has been discovered that curcumin shows anticarcinogenic, hepatoprotective, thrombo-suppressive, cardioprotective, antiarthritic, and anti-infectious properties. Curcumin has also shown neuroprotective properties in several preclinical studies for neurodegenerative diseases. This suggests that curcumin could be a worthy candidate for nutraceutical intervention in relation to neurodegenerative diseases [1]. Neurodegenerative diseases affect millions of people around the world. In addition, cognitive disorders caused by brain aging are becoming increasingly common, in part because the human life span has increased in recent years [2]. For these reasons, there is an urgent need to develop new and more effective approaches to combat these harmful conditions, both as a treatment and as prevention. While pharmaceutical treatments for neurodegenerative diseases are successful only in some patients, for others, researchers are looking for alternative options due to the lack of efficacy or intolerable side effects. For this reason, this review aimed to expose the main neuroprotective effects of vitD, alone and combined with curcumin, in order to illustrate how the use of natural dietary supplements can be useful in preventing or counteracting neuroinflammation progression. However, it is necessary to take into account that this field of research is still at the beginning of its path and many novelties await us if continue to explore this interesting molecule.

## 1. Vitamin D General Overview

In the present critical review, alongside the description of the most recent knowledge on the role of the essential micronutrient vitamin D (vitD) in various mechanisms underlying neurodegenerative disorders and other adverse effects, the potential effects of curcumin on the health of the central nervous system (CNS) are discussed, where curcumin is a nutraceutical that is extensively used in herbal medicine. Vitamin D has gained great fame as a nutritionally essential factor since the elucidation of vitamin D’s chemical structure revealed that it is a steroid hormone that is able to exert its effects through a specific receptor, which was discovered in the 1960s [3]. Vitamin D can either be ingested or synthesized in the skin [4,5], and to make it biologically active, the prohormone vitamin D is transported through the bloodstream to the liver, where it is metabolized [6]. 25-hydroxyvitamin D (25(OH)D3) is the major circulating metabolite of vitamin D in plasma and is essential for providing an index of a patient’s vitamin D nutritional status [7,8]. A successive metabolization of 25(OH)D3 generates the hormonally active form of vitamin D, namely, 1,25-dihydroxyvitamin D3 (1,25(OH)2D3), which is responsible for most of the biological actions of vitamin D [6]. It was assumed that the brain’s 1,25(OH)2D3 supply depends on the plasma concentration of 1,25(OH)2D3 (vitD) [9,10,11]. VitD works through two types of receptors: (i) the nuclear vitamin D receptor (VDR) to induce genomic action [12] and (ii) the putative membrane receptor MARRS (membrane-associated, rapid response steroid-binding) to induce non-genomic actions [13].

Maintaining an adequate plasma level of vitamin D can be a problem and vitamin D deficiency is more common than previously thought [14]. Natural food sources of vitamin D are uncommon; therefore, most people rely on skin production following safe exposure to sunlight. Exposure to sunlight is certainly the safest form of vitamin D supply and it could reduce the dependency on supplements [15]; however, many variables influence the amount of UV rays that reach the skin and its effectiveness. These include the time of day, season, latitude, altitude, clothing, use of sunscreen, pigmentation, and age. For this reason, especially in the elderly, exogenous administration through food supplements is necessary [16]. The U.S. National Academy of Science established a recommended daily intake for vitamin D of 15 μg/day (600 units/day) for people under the age of 70 and 20 μg/day (800 units/day) for people over the age of 70 [17]. Due to its broad therapeutic index, vitamin D toxicity is extremely rare. However, close attention should be paid to a prolonged excess of vitamin D intake, which could lead to hypercalcemia, hypercalciuria, and hyperphosphatemia, which are considered to be the initial signs of vitamin D intoxication. In particular, the impairment of the calcium/phosphate balance could lead to cardiovascular damage, such as arrhythmia, cardiac arrest, calcification of the vessels, and hypertension [18]. Recently, the localization of vitamin D3 25-hydroxylase and 25-hydroxyvitamin D3-1α-hydroxylase enzymes in the brain was demonstrated, confirming a local bioactivation of the vitamin D3 prohormone and its presence in the cerebrospinal fluid [19,20,21]. Furthermore, the local catabolism performed by vitamin D3 24-hydroxylase was found [22]. The fact that the CNS can locally perform both its activation and inactivation makes vitD a neurosteroid by definition [23]. Among the many functions that have been attributed to vitamin D, some concern the nervous system. A number of pleiotropic functions were recognized, such as maintaining healthy neuronal development, an adequate trophism of the adult brain, and a slow aging process [24]. VDRs are widely distributed throughout the embryonic and adult brain and appear most prominently in the neuroepithelium and proliferating zones in both rats [25,26,27,28,29] and humans [30]. Their presence has also been noted in neurons and glia of the human prefrontal and the cingulate cortices, thalamus, hypothalamus, cerebellum, substantia nigra, caudate, putamen, amygdala, and hippocampus [30,31]. Since the initial reports of Stumpf et al., on the presence of vitamin-D-specific nuclear binding in the brain and spinal cord [32,33], evidence has accumulated to suggest that both mRNA encoding the VDR and the protein itself are present in the nervous system. Thus, VDR gene expression has been demonstrated in neuronal and glial cells [34,35,36,37,38,39,40]. Expression of the VDR occurs early in the developing rodent brain at embryonic day (E) 11.5 and E12 in the rat dorsal root ganglion, spinal cord, and midbrain. Increasing levels of VDR expression throughout gestation coincides with increasing levels of apoptosis and decreasing levels of mitosis, and appears to be localized to the neuroepithelium and differentiating fields [41,42]. The sites of expression of VDR change during development, leading to the hypothesis that vitD may play a role in brain development [43]. For these reasons, the many functions that vitD exerts on the central nervous system have made it possible to hypothesize its use to counteract the mechanisms that lead to brain aging and neurodegenerative diseases.

## 2. Calcium Homeostasis in the Brain

Many calcium-binding proteins are present throughout the body, including the CNS [44,45]. It was reported that vitD in brain tissues or cells can modulate calbindin-D28K, parvalbumin, and calretinin [46,47]. These calcium-binding proteins are widely distributed in both the adult and fetal brain. In the adult brain, each of these proteins has a unique distribution and exhibits temporal patterns during development [48,49,50,51] and aging [52]. Calbindin-D28K is required for the normal signaling of synaptically evoked calcium transients [53] in synaptic plasticity [54], long-term potentiation (LTP) [55], memory formation [56,57], and possibly in the regulation of exocytosis (synaptic secretion of neurotransmitters) [58]. Furthermore, in Purkinje cells in the cerebellum, calbindin-D28kK appears to be directly involved in motor control [53,59], which could suggest a possible mechanism explaining motor deficits observed in vitamin-D-deficient rats. Calmodulin, is another important calcium-binding protein in the brain [60], although two studies reported that calcitriol treatment shifted the intracellular distribution of calmodulin [61]. In the brain, calmodulin is involved in neurotransmitter activity [62], N-methyl-D-aspartate (NMDA)-induced synaptic plasticity [63], and short-term plasticity [64]. Calcium/calmodulin-dependent protein kinase II (CAM kinase II) is highly concentrated in neurons and is believed to play a central role in a variety of brain functions, including learning and memory; it has been suggested that CAM kinase II is the molecular basis of long-term synaptic memory [65,66,67,68]. A recent report shows the down-regulation of the L-type voltage-sensitive calcium (Ca^2+^) channel in hippocampal neurons in the presence of vitD, which has been correlated with a neuroprotective effect against excitotoxic insults [69]. Another way in which vitD might mediate its neuroprotective effect is to induce the synthesis of Ca^2+^-binding proteins, such as parvalbumin [47].

## 3. Neurogenesis Is Stimulated by Vitamin D Metabolites and Some of Its Target Gene Products

VitD acts as a prodifferentiation hormone in many tissues. With reference to the brain, it is of interest that the increase in apoptotic cells and decrease in mitosis in the developing rat brain correlates with the appearance of VDRs [41]. Chronic treatment with calcitriol or its analogs increased the neurite outgrowth (and some other markers of differentiation) in human neuroblastoma cells in some significant experiments [70,71]. In rodents, vitD treatments stimulated neurite outgrowth in embryonic hippocampal explant cultures [72] and in a hippocampal progenitor cell line [73]. Adult neurogenesis is a very active area of research [74,75]. Adult stem cells are believed to be located in only two regions of the adult brain. One group of neural stem cells originates in the subventricular region of the forebrain and migrates to the olfactory bulb; the second group originates in the subgranular zone of the hippocampus and differentiates into neural and glial cells in the dentate gyrus [76,77]. In both cases, new neurons are local-circuit interneurons that link motor and sensory neurons [78]. There is a possible involvement of vitD in adult neurogenesis, with specific reference to the brain, where VDRs are widespread in both the olfactory bulb and the dentate gyrus in rodents [79] and were recently observed in the subventricular zone of the neonatal brain [80]. Furthermore, an increased density of hippocampal neurons was observed in some rat strains supplemented for 6–12 months with vitD (20 ng/rat, administered subcutaneously 5 times/week) [81].

### 3.1. Vitamin D and Neurotrophic Factors

The combination of in vitro, ex vivo, and animal model data provides evidence that vitD has a crucial role in neuronal proliferation, differentiation, neurotransmission, neuroplasticity, and neuroprotection. In addition, vitD levels correlate with the levels of several neurotrophic factors, including nerve growth factors (NGFs) and those of neurotrophins, which play crucial roles in the maintenance and growth of neurons [82,83]. In several cases, the neurotrophin production caused by vitD correlated with the neuroprotective effect, including the survival and migration of developing neurons in the brain [84,85]. Various studies have demonstrated that vitD can act on cells of the nervous system by up-regulating the synthesis of the NGF neurotrophin 3 (NT3), whereas it down-regulates neurotrophin 4 (NT4) [36,38,86] and glial cell line-derived neurotrophic factor (GDNF) [87]. NGF is present mainly in the hippocampus and neocortex, where it affects the growth and survival of developing neurons, neurotransmission, and synaptic plasticity [88]. Additional functions of vitD that are linked to neurotrophins include the enhancement of the synaptic transmission in the hippocampus by NT-3 [89] and its involvement in calcium signaling via NT4/5 [90]. Several studies on hippocampal explants and cultures treated with vitD have displayed increased NGF expression that is concomitant with a neurite outgrowth [36,86,91]. GDNF affects the survival and differentiation of dopaminergic cells and is present at relatively high levels in the developing striatum of the rat [92]. VitD administration leads to an increase in GDNF synthesis in both C6 glioma cells and the brain. In addition, in a stroke model, Wang and colleagues showed that VDH pre-treatment for 8 days can significantly increase the levels of GDNF and attenuate cortical infarction that is induced by a middle cerebral artery (MCA) ligation in rats [84,86].

### 3.2. Astrocytes as a Target of Neuroprotection

The mechanism behind astrocyte degeneration in age-related neurodegenerative disorders is unknown, although many different genetic abnormalities have been identified that can cause neurodegenerative disorders [93]. Furthermore, many studies in the literature provide indications as to the molecular and cellular factors that determine whether a particular neuron succumbs or resists an age-related disease [94]. The most vulnerable neurons are normally large with myelinated axons extending over long distances, from one region of the nervous system to another or from the central nervous system to peripheral targets [95,96,97,98,99]. This axon projection mechanism could be particularly linked to their vulnerability to aging, including high energy requirements, dependence on axonal transport (antegrade and retrograde) that support function and trophism, and a large cell surface that increases the risk of exposure of cells to toxic environmental conditions [100]. Normally, degeneration is often extended to subpopulations of neurons with a particular neurotransmitter phenotype. For example, amyotrophic lateral sclerosis (ALS) affects cholinergic motor neurons and striatal neurons containing GABA (γ-aminobutyric acid) and dopaminergic neurons, which are the most vulnerable in Huntington’s disease (HD) and Parkinson’s disease (PD), respectively [101]. Among the different neurotransmitters, glutamate could have an active and essential role in neuronal damage and death in all neurodegenerative disorders (see Section 3.3 on excitotoxicity below). Dysfunction and death of neurons negatively affect both the pre- and post-synaptic neurons with which they communicate [102].

### 3.3. Vitamin D3 and Neurodegenerative Diseases

The neuroprotective effect of vitD has recently been observed in the cognitive decline of aging rats [103], and it has been extensively studied in an animal model of multiple sclerosis (MS) and experimental allergic encephalomyelitis. The hormone prevents the onset and reversibly blocks the progression of clinical signs, but such a protective effect is absent in VDR knockout mice [104]. Some evidence implicates vitD as a candidate in influencing the susceptibility to a number of psychiatric and neurological diseases, such as schizophrenia, autism, PD, amyotrophic lateral sclerosis, epilepsy, and Alzheimer’s disease (AD), where the evidence is especially strong for MS [105,106]. Upon studying the effect of a vitD dietary restriction in the spinal cord and brain of ALS patients, there was a negative effect of vitD deficiency on the antioxidant capacity [107]. The effect of vitD might not be due exclusively to its neuro-immunomodulatory properties [105] since it has recently been reported that the hormone enhances neural stem cell proliferation and differentiation into neurons and oligodendrocytes, which are the myelinating cells of the central nervous system [108,109]. Neural stem cells constitutively express VDRs, which can be up-regulated by vitD. VitD regulates the expression of many AD-related genes. It attenuates amyloid-beta (Aβ)-peptide accumulation by stimulating phagocytosis of Aβ-peptide, probably by modulating the transcription of Toll-like receptors and cytokines, together with enhancing brain-to-blood efflux transport by increasing the P-glycoprotein expression [110]. Alterations in adult neurogenesis appear to be a common hallmark in different neurodegenerative diseases, including PD and AD [111]. Therefore, a hunt for possible molecules that are capable of stimulating neurogenesis is underway in order to develop new treatments for neurodegenerative disorders. Moreover, the combination of anti-neurodegenerative drugs with vitD supplementation might be useful. Indeed, the supplementation of the combination of nemantidine plus vitD has been shown to prevent cognitive decline more efficiently than that of the single compounds [112]. In addition, vitamin D exerts direct neuroprotective effects via the synthesis of Ca^2+^ ion binding proteins; this is important in neuronal function and in neuronal transmission. Proper levels of neuronal calcium are critical because their excess may result in the formation of reactive oxygen species (ROS) with consequent neuronal damage. Indeed, vitamin D levels are inversely associated with oxidative stress, which leads to neuronal apoptosis or necrosis [113]. VitD also affects neuroplasticity, a process in which neural synapses and pathways are adapted to the needs of environmental and behavioral demands by adjusting the brain to noxious stimuli diseases or environmental cues [114,115,116]. The VDRs in glial cells are involved in the uptake and release of neurotransmitters, including that of GABA neurotransmission within the motor cortex [117], which is the principal “brake” within the brain that affects muscle relaxation via the corticospinal neurons [118].

### 3.4. Vitamin D and Neuroinflammation

Concurrent administration of vitD with lipopolysaccharide (LPS) significantly inhibits inducible nitric oxide synthase (iNOS) expression in monocytes in the rat brain, suggesting that vitD can also help attenuate immune-induced oxidative damage in the CNS [119]. Lin et al. found a similar effect on zinc-induced toxicity in the CNS, where concurrent administration of vitD reduced apoptosis and oxidative damage [120]. An animal model of chronic relapsing experimental autoimmune encephalomyelitis (EAE), which is a model of MS, was used to study the effects of vitD [121]. VitD has been reported to be able to block the development of disease after its onset in both rats and mice [83,122] by providing an improvement in their condition, which was correlated with the inhibition of iNOS [123,124], CD4 antigen expression [122], and interleukin 12 (IL-12)-dependent TH1 cell development in the CNS [125]. VitD also increases the levels of transforming growth factor β (TGFβ) and IL-4, which were increased in a mouse model and are anti-inflammatory TH2 immune response cytokines [126]. In another EAE system, vitD significantly reduced acute inflammation and the levels of GFAP by inducing inflammatory cell apoptosis [127]. VitD has been observed to reduce the production of inflammatory cytokines tumor necrosis factor alpha (TNFα), IL-6, and nitric oxide (NO) in stimulated microglia [128]. VitD also appears to regulate the expression of N-myc, c-myc, PKC, and TGFβ in neuroblastoma cells [129], suggesting that it may affect neural cell growth in ways other than the well-established induction of NGF and its receptors [72,85,90,130].

### 3.5. Vitamin D3 and the Oxidative Defense System

An organism possesses defense mechanisms based on its antioxidants’ actions. Both enzymatic (e.g., superoxide dismutase (SOD), catalase, and glutathione reductase (GR)) or non-enzymatic (e.g., glutathione (GSH), melatonin, vitamins (A, C, E), and flavonoids) molecules play an important role in maintaining homeostasis and cell viability. Oxidative stress plays a key role in mitochondrial dysfunction, which will eventually lead to aging and the loss of function [131]. The mitochondrial free radical theory is one of the most studied hypotheses to explain this mechanism; it is based on the endogenous production of ROS and reactive nitrogen species (RNS) and their harmful effects on the mitochondria [132,133]. These species react with lipids, proteins, and nucleic acids, causing oxidative damage that leads to a progressive decline in cellular functions [107,134]. A study on the mechanisms of vitD protection against methamphetamine-induced reductions in brain levels of dopamine and serotonin showed that they include an increase in the levels of glutathione, along with the inhibition of iNOS production, both of which could reduce the toxicity caused to dopamine and serotonin neural terminals [135]. VitD can regulate lipid peroxidation as a form of neuroprotection through inducing the synthesis of parvalbumin, a Ca^2+^ binding protein, which helps with maintaining the cellular calcium homeostasis and eventually lowers lipid peroxidation [136], as well as through promoting the expression of calcium buffering proteins calbindin-D28K and calbindin-d9K, which reduce the malonaldehyde (MDA) levels [137,138]. During a CNS injury, there is a high release rate of nitric oxide (NO) that reacts with superoxide and eventually produces nitrogenous species, especially peroxynitrite (ONOO^−^), which is a marker in the spinal cord of ALS patients that inactivates SOD2 through dityrosine formation [139]. The reduction of SOD2 activity is through the post-translational impact of vitD on nuclear factor κB (NF-κB), as activated microglia in ALS use the NF-κB pathway to induce mitochondrial dysfunction inhibition of SOD2 and motor neuron death [140,141]. In addition, a cross-sectional study on type 2 diabetes mellitus patients was performed to investigate the correlation between vitD status and the antioxidant profile in diabetic patients compared to healthy groups, where they were followed up for fasting serum levels of 25-OH-D, calcium, phosphorus, parathyroid hormone, glucose, HbA1C, insulin, total antioxidant capacity (TAC), SOD, GR, and glutathione peroxidase (GSH-Px). These findings have indicated an inverse relationship between the serum levels of 25-OH-D and the activities of both GSH-PX and GR, along with a positive correlation with SOD in diabetic patients. Meanwhile, in healthy subjects, there is an inverse relation between 25-OH-D levels and SOD and GSH-PX levels, and a positive correlation with GR activities. The positive association between the serum level of 25-OH-D and GR is due to the role of GSH in maintaining the intracellular redox balance. VitD acts by increasing the activity of GR and decreasing the GSH-PX activity in healthy subjects to enhance the GSH pool [142]. VitD has also been reported to inhibit the synthesis of iNOS [119,123], which is an enzyme induced in CNS neurons and non-neuronal cells during various insults or diseases, such as ischemia, Alzheimer’s disease, Parkinson’s disease, AIDS, infections, multiple sclerosis, and EAE. iNOS produces nitric oxide, one of the biological effects of which is to damage both neurons and oligodendrocytes when it is produced at high levels [143,144]. VitD has also been reported to up-regulate γ-glutamyl transpeptidase activity and the expression of the corresponding gene in rat brains and reduce the nitrite production in LPS-stimulated primary rat astrocyte culture because γ-glutamyl transpeptidase is largely involved in the glutathione cycle of the brain in the crosstalk between astrocytes and neurons [145,146,147]. In conclusion, regarding the oxidative mechanism involving vitD, treatments with vitamin D3 and other compounds that are capable of improving its effect could be a promising remedy for the treatment of neurodegenerative diseases during aging.

### 3.6. Curcumin General Overview

Curcumin is the principal curcuminoid of the popular Indian spice turmeric, and its main active ingredient is obtained from the rhizome of Curcuma longa *Linn*. Curcumin acts as a free radical scavenger and antioxidant, inhibiting lipid peroxidation [148] and oxidative DNA damage. Moreover, it is a particularly potent immuno-regulatory agent that can modulate the activation and function of T-cells, B-cells, neutrophils, natural killer cells, and macrophages [149]. Curcumin treatment effectively inhibits the activation of microglial cells by diminishing the production of nitric oxide [150] and reducing the secretion of pro-inflammatory cytokines, such as IL1β, IL6, and TNF [151]. Recent experiments have also demonstrated that curcumin protects dopaminergic neurons against microglia-mediated neurotoxicity [152], limits brain inflammation [153], and rescues retinal cells from stress-induced cell death [154]. The suppressive effect of curcumin is thought to involve regulating the JAK–STAT inflammatory signaling in activated microglia [155]. A recent report has revealed that curcumin reduces the amyloid-β-stimulated inflammatory responses in primary astrocytes. The deleterious effects of amyloid-β, such as the increased expression of COX-2 and glial fibrillary acidic protein and the decreased peroxisome proliferator-activated receptor gamma (PPARγ), were attenuated using pretreatment with curcumin [156]. Accumulating cell culture and animal model data show that curcumin is a strong candidate for use in the prevention or treatment of major disabling age-related neurodegenerative diseases, such as AD, PD, and strokes [157]. Furthermore, it was shown that curcumin can down-regulate the expression of IL-6, TNF, and various other chemokines [149]. Moreover, it has subsequently been shown that curcumin down-regulates the expression of the NF-κB-regulated gene products, such as COX-2, TNF, 5-LOX, IL-1, IL-6, IL-8, MIP-1α, adhesion molecules, c-reactive protein (CRP), and CXCR-4 [158,159,160,161].

### 3.7. Neuroprotective Effects of Curcumin

Curcumin has also been reported to bind to COX-2 and 5-LOX and inhibit their activity [162]. In a study on the protective effect of curcumin on dopaminergic neurons from apoptosis in an MPTP mouse model of PD, Curcumin markedly ameliorated the loss of dopaminergic axons in the striatum as well as the death of dopaminergic neurons, further mechanistic studies demonstrated that curcumin inhibits MPTP-induced hyperphosphorylation of c-Jun N-terminal kinase (JNK); it also prevents the degeneration of nigrostriatal neurons by inhibiting the dysfunction of mitochondria through abolishing the hyperphosphorylation of JNKs induced by MPTP [163]. Moreover, curcumin’s inhibitory effect on NO and PGE2 production was associated with decreased iNOS and COX-2 expressions in LPS-stimulated BV2 microglia, where the results of Western blot analysis revealed that iNOS and COX-2 protein levels were undetectable in unstimulated BV2 microglia [151]. A study was done to evaluate the neuroprotective effects of curcuminoids on B35 and SH-SY5Y neuroblastoma cells after subjecting the cells to hydrogen peroxide, followed by curcumin at different concentrations of 5, 10, and 20 μM before and after being damaged. B35 neuroblastoma cells showed an increase in cell viability when treating cells with curcumin before and after the induced damage. Meanwhile, the SH-SY5Y neuroblastoma cells showed an increase in their viability only after the curcumin treatment. Caspase-3 and caspase-9, which are important apoptosis mediators, were examined, where the curcumin inhibited the caspase-3 in a concentration-dependent manner but not caspase-9. These findings suggest that curcumin is a neuroprotectant and an anti-apoptotic agent that acts through the inhibition of caspase-3; hence, it can be introduced as a potential agent to treat or prevent neurodegenerative diseases [164]. The results of a study performed by Yang and his colleagues show that curcumin at a 10 µM concentration had a neuroprotective effect on midbrain dopaminergic neurons; increased the dopamine uptake; decreased the expression of proinflammatory cytokines, such as nitric oxide (NO), prostaglandin E2 (PGE2), IL-1β, and TNF-α; inhibited the transcription of NF-κB and activator protein-1 [153]. However, another group of researchers has identified a possible molecular target for curcumin that may be involved in SH-SY5Y dopaminergic neuron protection during MPP+-induced cytotoxicity (3 mM MPP+). This mechanism involves JNK pathway activation and caspase-3 cleavage, preventing neuronal death [165]. A study of another mechanism for curcumin in apoptosis investigated the effects of curcumin in appoptosin-induced apoptosis in SH-SY5Y cells, where appoptosin (SLC25A38) is a pro-apoptotic protein that is up-regulated in Alzheimer’s disease (AD) brains and promotes the pathological progress. Through pretreating SH-SY5Y cells with curcumin, then transfecting with appoptosin, followed with detecting apoptotic cells with Annexin V staining analysis using flow cytometry, the expression of cleaved caspase-3, appoptosin, and heme oxygenase-1 (HO-1) were examined and the intracellular ROS level was measured to detect the mitochondrial membrane potential. The results showed an overexpression of appoptosin, which was accompanied by reduced HO-1 expression, ROS overproduction, and mitochondrial potential impairment. However, pretreating with curcumin (2.5–20 μmol/L) in a dose-dependent manner attenuated all these pathological changes in appoptosin-transfected SH-SY5Y cells [166].

### 3.8. Effectiveness of Vitamin D Dietary Supplements in a Clinical Study

Starting from 2011, many systematic reviews and meta-analyses have reported useful data for the development of evidence-based clinical practice guidelines [167] on vitamin D supplementation. Several systematic epidemiologic studies that were based on the association between vitamin D and cognitive decline, dementia, and neurodegenerative conditions have been published [168]. However, the data show discordant results since some studies claim a positive role of vitamin D in the brain, while others even indicate adverse effects. For example, a meta-analysis reported by Suzuki et al. [169] showed that older adults with PD taking supplemental vitamin D in randomized controlled trials showed beneficial muscle effects in terms of strength and balance [169]. Nonetheless, it cannot be distinguished whether vitamin D supplementation specifically delays the progression of PD or whether it just nonspecifically improves muscle strength and balance in older adults. In another study, long-stay patients with baseline 25(OH)D < 40 nmol/L were treated with 9000 IU vitamin D2 for 8–40 weeks or a placebo (lactose tablets). No significant difference in the single cognitive measure (mental assessment score) was found between groups [170]. Barnard and Colon-Emeric suggested that cognitive function, as measured using the Mini-Mental State Examination (MMSE), was not associated with the 25(OH)D concentration, although their conclusions were based on whether the relationship between vitamin D and cognitive test scores in the original studies was statistically significant [171]. Another trial with 4143 participants compared vitamin D3 (400 IU/day) and calcium supplements to a placebo. Rutjes et al. [172] found low- to moderate-certainty evidence of no effect of vitamin D3 and calcium supplements at any time point during 10 years on the overall cognitive function (MD after a mean of 7.8 years = − 0.1 MMSE points, 95% CI − 0.81 to 0.61) or the incidence of dementia (HR = 0.94, 95% CI = 0.72 to 1.24). Based on this result, a pilot study with 60 participants used a higher dose of vitamin D3 (4000 IU on alternate days) and found preliminary evidence that this dose probably had no effect on cognitive function over six months [172]. Therefore, they found no evidence that a vitamin D supplementation strategy for cognitively healthy adults had a meaningful effect on cognitive decline or dementia, although the evidence does not permit definitive conclusions. Interventional trials investigated whether vitamin D supplementation can beneficially influence the brain disease course, but the trials have been methodologically fairly heterogeneous, and study designs have not always been appropriate [173]. Some studies were not longitudinal and could have confounding factors; therefore, the final result could be unclear. Due to this scenario, we investigated even further because vitamin D research has gained increased attention in recent times due to its roles beyond bone health and calcium homeostasis.

A randomized controlled double-blind placebo trial investigated the effect of vitamin D on cognition in healthy elderly subjects above 65 years of age. The vitamin-D-supplemented group (supplement contained 4.0 μg (160 IU) of vitamin D and other trace elements) had a better cognitive performance (*p* < 0.01) in the different cognitive tests (seven cognitive tests, including the MMSE) compared to the placebo group [174]. Another prospective pre–post interventional study, which included 80-year-old subjects from a memory clinic, found that those who received oral vitamin D3 supplementation (800 IU per day or 100,000 IU per month) experienced improved global cognition and executive functioning abilities over a 16-month follow-up period compared to controls [175]. Bailon et al. [176], in its systematic review of meta-analysis cognition data (using the MMSE) provided suggestive evidence of a possible relationship between vitD and cognitive decline, but the nature of this relationship remained unclear. This discrepancy may be a function of the type of cognitive measure used. Little is known about the function of vitamin D in relation to the different cognitive domains. To date, no treatment study has examined this question where both vitamin D and cognition were measured over a sufficient period in a large at-risk population. In particular, Balion et al. [176] included data from eight cross-sectional and case-control studies with a total of 2749 participants, comparing MMSE scores between participants who were given a 25(OH)D concentration < 50 nmol/L and those who were given a 25(OH)D ≥ 50 nmol/L. This review demonstrated a higher average MMSE score with a higher vitamin D concentration (average difference 1.2; 95% CI 0.5–1.9) with a significant heterogeneity (I^2^ = 0.65) [168]. Therefore, it is clear that the difference between the studies not only concerns the method applied but also the dosages. It appears from these studies that at low doses, the beneficial effects are observable, while at high doses, these effects become nil. In summary, vitamin D supplementation should be encouraged in the elderly and in AD patients to combat possible cognitive decline; meanwhile, a huge body of evidence emphasizes the importance of vitamin D as a supplement for slowing brain aging. In addition, based on statistical and clinical results, vitamin D treatment may be a safe option for brain supplements due to its potential synergistic benefits if used at a correct dosage and in combination with other molecules. For example, in a pilot study, Annweiler et al. [177] explored the combination of vitD and memantine to improve cognitive parameters in AD patients. Memantine, which is a Japanese drug approved by the FDA (Food and Drug Administration) to treat moderate-to-severe AD, is able to exert its therapeutic effect by acting as a low to moderate affinity, non-competitive NMDA receptor antagonist that blocks the Ca^2+^ ion flux [178]. Treatment with memantine plus vitamin D was associated with improvement in the MMSE score compared to memantine or vitamin D alone. Patients with AD who took memantine plus vitamin D for 6 months had a statistically and clinically relevant gain in cognition, supporting the hypothesis of the possible synergistic and potential benefits of the combination. This study confirmed a possible beneficial role of vitD when used correctly and its possible beneficial interaction with other molecules.

### 3.9. Interaction between Vitamin D3 and Curcuminoids

**Figure 1:**
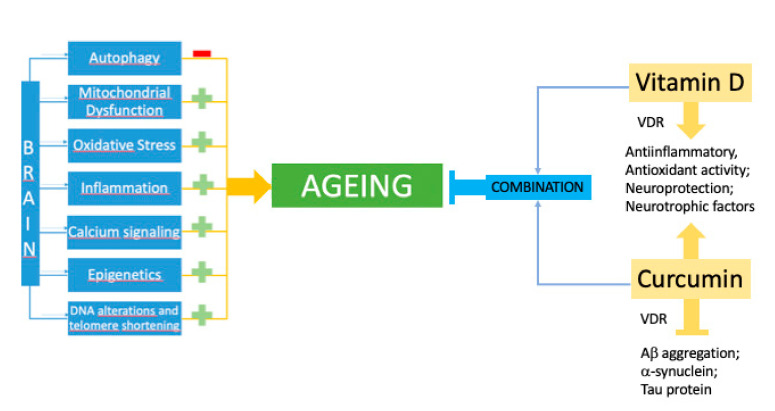
Schematic representation of the combined effects of vitD and curcumin to slow down brain aging. VDR: vitamin D receptor.

The results of many studies may be relevant to the use of curcuminoids and vitamin D3 in the prevention of neurodegenerative diseases, which are substances that work via antioxidant and anti-inflammatory mechanisms ([179] Figure 1). Indeed, morbidities of aging have been related to defective functions of both T cells and macrophages leading to brain amyloidosis and inflammation [180]. “Inflammaging” may be associated with an increase in incompetent memory T cells and inflammatory cytokines produced by macrophages, whereas the defective clearance of amyloid-beta Aβ1–42 may be related to the defective transcription of immune genes necessary for phagocytosis, β-1,4-mannosyl-glycoprotein, 4-β-N-acetylglucosaminyltransferase, and Toll-like receptors. The approaches used for re-balancing Aβ immunity and inflammation are being pursued in animal models and the peripheral blood mononuclear cells of patients [179,181,182,183]. Increased inflammation may represent the “first hit” and defective transcription of immune genes represent the “second hit” in the pathogenesis of AD.

Gagliardo et al. conducted studies that showed that treatment with curcumin may decrease the predisposition of Aβ to accumulate in cells. Although the role of Aβ_1-42_ as a potential causative agent in the pathophysiology of neurodegenerative disease has been documented extensively, the exact molecular mechanisms and their biochemical effect on brain tissues are not very clear [179,183]. An in vitro experiment by Alamro et al. on primary neuronal cultures investigated the effect of Aβ_1-42_ on the primary neuronal cells and the role of vitamin D3 or curcumin used as a natural therapy for AD treatment. It was confirmed that the treatment of primary neuronal cells with Aβ_1-42_ could cause elevated levels of oxidative stress, which is depicted by an increased amount of lipid peroxidation products and decreased levels of antioxidant enzymes [184]. According to this, curcumin treatment produced a decrease in intracellular Aβ aggregates, an increase of phagocytosis (e.g., the MGAT3 pathway), and a reduction of inflammation. Results of the treatment of PBMCs (peripheral blood mononuclear cells) from AD patients with curcuminoids demonstrated the curcuminoids’ ability to act on cell susceptibility to Aβ accumulation. Data showed that *NF-κB* and *BACE1* were decreased after treatment with curcuminoids. In addition, the decrease in *NF-κB* led to the suppression of the inflammatory cascade, which is one of the major pathways in aging pathology [185]. In this context, it is necessary to emphasize the role of vitamin D3 as a transcription regulator of genes of the innate immune system [186]. It has been hypothesized that vitamin D3 possesses immunostimulant effects and also induces the phagocytosis and degradation of Aβ via monocyte/macrophage maturation [182,187]. We suggest that increased MGAT3 and VDRs inhibit Aβ accumulation and decrease inflammation. Moreover, in most systems, vitamin D inhibits the activation of NF-κB [188]. VDR stimulation induced by curcuminoids could shift this balance to and increase the inhibitory activity of VDRs on NF-κB [189]. Another study on primary cortical neuronal cells demonstrated that Aβ_1-42_ caused a significant reduction in mitochondrial health or mitophagy, but treatment with vitamin D3 and curcuminoids could overturn this outcome [183]. The oxidative state of the primary cortical neuronal cells is one of the earliest hallmarks of neurodegenerative diseases [190]. Normally, cells have an antioxidant mechanism, such as GSH and antioxidant enzymes, catalase, and SOD, that can quench free radicals [191,192], but this mechanism is dysregulated during aging. As said before, lipid peroxidation is the main contributor to free-radical-mediated damage to the neuronal membrane; it can also produce some secondary oxidation products that are capable of causing additional cellular damage. These highly reactive electrophilic aldehydes are MDA and 4-hydroxy-2-nonenal (HNE) [193]. Alamro et al. [183] showed that a significant amount of MDA in the Aβ_1–42_ treated cultures produced high levels of these aldehydes, but the presence of vitamin D3 and curcumin in the culture categorically reduced its formation [190]. The synergistic effect of vitamin D3 and curcumin significantly increased the catalase enzyme expression. These results further reiterated the beneficial therapeutic effect of both vitamin D3 and curcumin in the treatment of AD. The combined effect of vitamin D3 and curcumin has also been reported to accelerate the clearance of β-amyloid by activating the immune cells [183]. These results have been confirmed by SOD enzyme activity analysis, where the activity was significantly reduced with the treatment of Aβ_1–42_ to the cells, but the synergistic effect of both vitamin D3 and curcumin has a positive effect on up-regulating SOD enzyme activity. Finally, the neuroprotective action of vitamin D3 on neuronal tissue is due to the up-regulation of several genes involved in alleviating oxidative stress, such as reduced GSH and NGF [114]. In recent years, the targeted delivery of NGF to the affected areas of Basal Forebrain-Like Cholinergic Neurons (BFCN) as a potential therapy for the AD patients has gained support due to the regenerative effect of NGF on BFCN [194]. During aging, there is a significant loss of NGF; however, this effect was neutralized in the presence of vitamin D3 or curcumin. Earlier studies have indicated the role of vitamin D3 in the regulation and secretion of NGF and GDNF [195]. Curcumin has also been shown to initiate proliferation and improve the health of neurite growth and synaptic activity [196].

### 3.10. A New Possible Treatment Approach to Support Brain Health

The data shown so far demonstrate that there are several functions played by vitamin D and curcumin, especially in neuroprotection and maintaining brain health. Furthermore, these substances could play an important role in the early, mild stages of neurodegenerative diseases and in cognitive impairment. Therefore, the question arose about the use of such compounds regarding combined treatment, whether there is a synergistic effect, and whether they are safe to use in such a combination. In a study published in 2015, in which a scopolamine-hydrobromide-induced Alzheimer’s disease rat model was used to reduce the effective action at the synapse by antagonizing muscarinic acetylcholine receptor without changing the concentration of acetylcholine to produce a stage of memory impairment, animals were subjected to a drug treatment schedule for 27 days, where there was a scopolamine group, a scopolamine–curcumin group, a scopolamine–vitamin D group, and a scopolamine–donepezil group. All groups underwent behavioral tests, namely, the rectangular maze and locomotor activity tests, followed by histological analysis for cellular degeneration to determine whether there was amelioration in this stage after treatment, and immunoblotting procedures were used to detect the expression of modified microtubule-associated tau protein. Results show that scopolamine treatment has led to a decrease in transfer latency as a result of significant memory loss, which was obvious in behavioral tests, where a large number of degenerated cells was observed in histological imaging along with a significant presence of abnormal tau protein. In contrast, treatment groups had an ameliorated transfer latency regarding locomotion, indicating improved memory, as seen from the behavioral test results, where the histological examination showed equal cell numbers and similar cell morphology compared to the control groups, which proved the presence of memory regions. Furthermore, there was a strong reduction in tau phosphorylation normalized to β-actin. From these results, they concluded the potential of curcumin and vitD to reverse some cognitive and memory impairment within the same AD-induced model [197].

## 4. Conclusions

Based on all the studies previously described on vitD and curcumin, which have described the function of these molecules and the possible mechanisms activated in different neurodegeneration models, it could be interesting to consider the administration of vitD and curcumin as a supplement in a continuous way. Although it is good to remember that correct and safe exposure to sunlight could reduce the dependence on exogenous vitamin D supplementation, there are conditions in which the use of supplements is necessary. In the future, it would be useful to explore the existence of a synergistic effect for these compounds when administered in a combined preparation. Further studies will be needed in order to test their neuroprotective and therapeutic abilities to maintain a healthy brain during aging or to prevent neurodegeneration.
